# Poly[[tetra­aqua­(μ_3_-naphthalene-1,6-disulfonato-κ^4^ *O* ^1^:*O* ^6^,*O* ^6′^:*O* ^6′′^)strontium(II)] monohydrate]

**DOI:** 10.1107/S1600536811047593

**Published:** 2011-11-16

**Authors:** Shan Gao, Seik Weng Ng

**Affiliations:** a Key Laboratory of Functional Inorganic Material Chemistry, Ministry of Education, Heilongjiang University, Harbin 150080, People’s Republic of China; b Department of Chemistry, University of Malaya, 50603 Kuala Lumpur, Malaysia; c Chemistry Department, Faculty of Science, King Abdulaziz University, PO Box 80203 Jeddah, Saudi Arabia

## Abstract

In the crystal structure of the polymeric title compound, {[Sr(C_10_H_6_O_6_S_2_)(H_2_O)_4_]·H_2_O}_*n*_, the naphthalene-1,6-disulfonate dianion uses one –SO_3_ unit to *O*,*O*′-chelate to an Sr^II^ cation and its third O atom to bind to another Sr^II^ cation. The other –SO_3_ unit binds to yet another Sr^II^ atom. The four coordinated water mol­ecules are monodentate but one is disordered over two positions in a 1:1 ratio. The μ_3_-bonding mode of the dianion generates a polymeric three-dimensional network; the network is consolidated by O—H⋯O hydrogen bonds. The Sr^II^ cation exists in an undefined eight-coordinate environment.

## Related literature

For a review of metal arene­sulfonates, see: Cai (2004 ▶). For a related strontium naphthalene­disulfonate, see: Cai *et al.* (2001 ▶). 
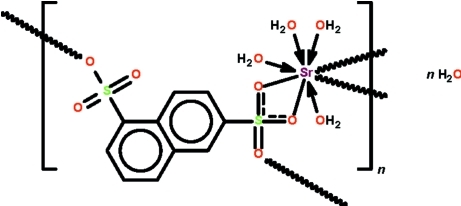


## Experimental

### 

#### Crystal data

[Sr(C_10_H_6_O_6_S_2_)(H_2_O)_4_]·H_2_O
*M* *_r_* = 463.97
Orthorhombic, 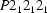

*a* = 7.1067 (16) Å
*b* = 14.080 (4) Å
*c* = 16.745 (6) Å
*V* = 1675.6 (9) Å^3^
*Z* = 4
Mo *K*α radiation
μ = 3.52 mm^−1^
*T* = 293 K
0.22 × 0.17 × 0.15 mm

#### Data collection

Rigaku R-AXIS RAPID IP diffractometer
Absorption correction: multi-scan (*ABSCOR*; Higashi, 1995 ▶) *T*_min_ = 0.511, *T*_max_ = 0.620
16056 measured reflections
3786 independent reflections
3497 reflections with *I* > 2σ(*I*)
*R*_int_ = 0.030

#### Refinement

*R*[*F* ^2^ > 2σ(*F* ^2^)] = 0.024
*wR*(*F* ^2^) = 0.057
*S* = 1.02
3786 reflections
220 parameters
15 restraints
H-atom parameters constrained
Δρ_max_ = 0.36 e Å^−3^
Δρ_min_ = −0.29 e Å^−3^
Absolute structure: Flack (1983 ▶), 1584 Friedel pairs
Flack parameter: −0.017 (4)

### 

Data collection: *RAPID-AUTO* (Rigaku, 1998 ▶); cell refinement: *RAPID-AUTO*; data reduction: *CrystalClear* (Rigaku/MSC, 2002 ▶); program(s) used to solve structure: *SHELXS97* (Sheldrick, 2008 ▶); program(s) used to refine structure: *SHELXL97* (Sheldrick, 2008 ▶); molecular graphics: *X-SEED* (Barbour, 2001 ▶); software used to prepare material for publication: *publCIF* (Westrip, 2010 ▶).

## Supplementary Material

Crystal structure: contains datablock(s) global, I. DOI: 10.1107/S1600536811047593/xu5382sup1.cif

Structure factors: contains datablock(s) I. DOI: 10.1107/S1600536811047593/xu5382Isup2.hkl

Additional supplementary materials:  crystallographic information; 3D view; checkCIF report

## Figures and Tables

**Table 1:** Selected bond lengths (Å)

Sr1—O1	2.737 (2)
Sr1—O2	2.721 (2)
Sr1—O3^i^	2.583 (2)
Sr1—O4^ii^	2.5352 (19)
Sr1—O1*W*	2.641 (2)
Sr1—O2*W*	2.562 (2)
Sr1—O3*W*	2.500 (2)
Sr1—O4*W*	2.585 (14)

Symmetry codes: (i) 
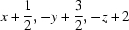
; (ii) 
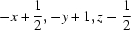
.

**Table 2:** Hydrogen-bond geometry (Å, °)

*D*—H⋯*A*	*D*—H	H⋯*A*	*D*⋯*A*	*D*—H⋯*A*
O1w—H12⋯O2^iii^	0.84	2.25	2.809 (3)	124
O2w—H21⋯O5^iv^	0.84	2.03	2.793 (3)	151
O2w—H22⋯O5w^v^	0.84	1.95	2.763 (3)	164
O3w—H31⋯O6^vi^	0.84	2.09	2.829 (3)	147
O3w—H32⋯O5w^v^	0.84	1.99	2.754 (3)	151
O5w—H51⋯O6^ii^	0.84	2.06	2.874 (3)	163
O5w—H52⋯O1w	0.84	2.02	2.831 (3)	160

Symmetry codes: (ii) 
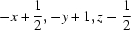
; (iii) 
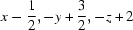
; (iv) 
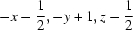
; (v) 
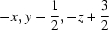
; (vi) 
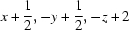
.

**Fig. 1.**
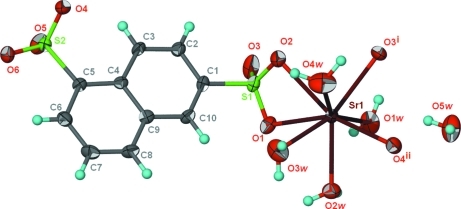
Thermal ellipsoid plot (Barbour, 2001) of a fragment of polymeric Sr(H2O)4(C10H6O6S2).H2O at the 50% probability level; hydrogen atoms are drawn as spheres of arbitrary radius.

## supplementary crystallographic information

### Comment

A review of metal arenesulfonates that are synthesized in aqueous medium explains the reasons for the ability of the ions to form stable metal-organic frameworks owing to multiple coordination modes of the sulfonate –SO_3_ groups (Cai, 2004). Among the divalent metal derivatives, the strontium system has been less studied (Cai *et al.*, 2001). In the crystal structure of Sr(H_2_O)_4_(C_10_H_6_O_6_S_2_)^.^H_2_O, the C_10_H_6_O_6_S_2_^2-^ dianion uses one –SO_3_ unit to *O*,*O*'-chelate to an Sr^II^ atom and its third O atom to bind to another Sr^II^ atom. The other –SO_3_ unit binds to yet another Sr^II^ atom (Scheme I, Fig. 1). T; the four coordinated water molecules are monodentate but one is disordered over two positions in a 1:1 ratio. The *µ*_3_ bonding mode of the dianon generates a polymeric three-dimensional network; the network is consolidated by O–H···O hydrogen bonds (Table 1). The Sr atom exists in an undefined eight-coordinate environment.

### Experimental

Strontium nitrate (1 mmol) and sodium naphthalene-1,6-disulfonate (1 mmol) were dissolved in water (10 ml). The solution was filtered and set aside; yellow crystals were isolated from the filtrate after several days.

### Refinement

Carbon-bound H-atoms were generated geometrically and were included in the riding model approximation [C—H 0.93 Å, *U*, 1.2*U*_eq_(C)]. The water H-atoms were placed in calculated positions [O—H 0.84 Å, *U* 1.5*U*_eq_(O)] on the basis of hydrogen bonding interactions; however, only some are involved and others are not.

One of the water molecules is disordered over two positions in a 1:1 ratio.

### Crystal data

**Table:**

[Sr(C_10_H_6_O_6_S_2_)(H_2_O)_4_]·H_2_O	*F*(000) = 936
*M**_r_* = 463.97	*D*_x_ = 1.839 Mg m^−^^3^
Orthorhombic, *P*2_1_2_1_2_1_	Mo *K*α radiation, λ = 0.71073 Å
Hall symbol: P 2ac 2ab	Cell parameters from 14836 reflections
*a* = 7.1067 (16) Å	θ = 3.1–27.1°
*b* = 14.080 (4) Å	µ = 3.52 mm^−^^1^
*c* = 16.745 (6) Å	*T* = 293 K
*V* = 1675.6 (9) Å^3^	Prism, yellow
*Z* = 4	0.22 × 0.17 × 0.15 mm

### Data collection

**Table:**

Rigaku R-AXIS RAPID IP diffractometer	3786 independent reflections
Radiation source: fine-focus sealed tube	3497 reflections with *I* > 2σ(*I*)
graphite	*R*_int_ = 0.030
ω scan	θ_max_ = 27.1°, θ_min_ = 3.1°
Absorption correction: multi-scan (*ABSCOR*; Higashi, 1995)	*h* = −8→9
*T*_min_ = 0.511, *T*_max_ = 0.620	*k* = −16→18
16056 measured reflections	*l* = −21→21

### Refinement

**Table:**

Refinement on *F*^2^	Secondary atom site location: difference Fourier map
Least-squares matrix: full	Hydrogen site location: inferred from neighbouring sites
*R*[*F*^2^ > 2σ(*F*^2^)] = 0.024	H-atom parameters constrained
*wR*(*F*^2^) = 0.057	*w* = 1/[σ^2^(*F*_o_^2^) + (0.0282*P*)^2^] where *P* = (*F*_o_^2^ + 2*F*_c_^2^)/3
*S* = 1.02	(Δ/σ)_max_ = 0.001
3786 reflections	Δρ_max_ = 0.36 e Å^−^^3^
220 parameters	Δρ_min_ = −0.29 e Å^−^^3^
15 restraints	Absolute structure: Flack (1983), 1584 Friedel pairs
Primary atom site location: structure-invariant direct methods	Flack parameter: −0.017 (4)

### Fractional atomic coordinates and isotropic or equivalent isotropic displacement parameters (Å^2^)

**Table:**

	*x*	*y*	*z*	*U*_iso_*/*U*_eq_	Occ. (<1)
Sr1	0.17295 (3)	0.709854 (16)	0.858456 (15)	0.02516 (7)	
S1	−0.05018 (9)	0.60924 (5)	1.00949 (4)	0.03027 (15)	
S2	0.03060 (8)	0.15903 (4)	1.19980 (4)	0.02706 (14)	
O1	−0.1183 (2)	0.61336 (14)	0.92809 (12)	0.0384 (5)	
O2	0.1341 (3)	0.65457 (14)	1.01330 (13)	0.0419 (5)	
O3	−0.1813 (3)	0.64691 (14)	1.06765 (13)	0.0512 (6)	
O4	0.1872 (3)	0.20770 (14)	1.23766 (11)	0.0367 (4)	
O5	−0.1493 (3)	0.18114 (15)	1.23623 (12)	0.0435 (5)	
O6	0.0641 (3)	0.05717 (13)	1.19636 (13)	0.0355 (4)	
O1W	−0.0894 (3)	0.84232 (15)	0.86687 (16)	0.0516 (6)	
H11	−0.0628	0.8806	0.9036	0.077*	
H12	−0.1939	0.8169	0.8764	0.077*	
O2W	−0.0715 (3)	0.68007 (15)	0.74884 (13)	0.0430 (5)	
H21	−0.1701	0.7106	0.7592	0.065*	
H22	−0.0964	0.6218	0.7467	0.065*	
O3W	0.2189 (3)	0.54239 (15)	0.81275 (15)	0.0550 (6)	
H31	0.3323	0.5340	0.8004	0.082*	
H32	0.1506	0.5320	0.7728	0.082*	
O4W	0.518 (2)	0.6538 (11)	0.8714 (6)	0.069 (2)	0.50
H41	0.5845	0.6977	0.8902	0.103*	0.50
H42	0.5236	0.6071	0.9026	0.103*	0.50
O4W'	0.506 (2)	0.6686 (11)	0.9022 (6)	0.069 (2)	0.50
H43	0.5283	0.6115	0.8912	0.103*	0.50
H44	0.5160	0.6770	0.9517	0.103*	0.50
O5W	0.0879 (3)	0.98623 (17)	0.77588 (17)	0.0621 (7)	
H51	0.1881	0.9625	0.7577	0.093*	
H52	0.0479	0.9502	0.8123	0.093*	
C1	−0.0216 (3)	0.48828 (18)	1.03469 (16)	0.0273 (6)	
C2	−0.0014 (4)	0.4641 (2)	1.11612 (16)	0.0311 (6)	
H2	0.0027	0.5116	1.1547	0.037*	
C3	0.0120 (3)	0.37121 (17)	1.13822 (17)	0.0307 (5)	
H3	0.0240	0.3561	1.1921	0.037*	
C4	0.0081 (3)	0.29677 (19)	1.08087 (14)	0.0242 (5)	
C5	0.0181 (3)	0.19898 (19)	1.09993 (15)	0.0257 (5)	
C6	0.0120 (3)	0.1312 (2)	1.04032 (17)	0.0328 (6)	
H6	0.0171	0.0671	1.0536	0.039*	
C7	−0.0016 (4)	0.1581 (2)	0.96025 (18)	0.0371 (7)	
H7	−0.0033	0.1118	0.9206	0.045*	
C8	−0.0124 (4)	0.2518 (2)	0.93974 (17)	0.0346 (6)	
H8	−0.0236	0.2688	0.8863	0.042*	
C9	−0.0066 (3)	0.32300 (18)	0.99881 (15)	0.0260 (5)	
C10	−0.0232 (3)	0.42029 (19)	0.97758 (16)	0.0290 (6)	
H10	−0.0353	0.4374	0.9242	0.035*	

### Atomic displacement parameters (Å^2^)

**Table:**

	*U*^11^	*U*^22^	*U*^33^	*U*^12^	*U*^13^	*U*^23^
Sr1	0.02683 (11)	0.02339 (11)	0.02527 (12)	−0.00045 (10)	−0.00087 (10)	0.00222 (10)
S1	0.0361 (3)	0.0258 (3)	0.0289 (4)	0.0041 (3)	0.0054 (3)	0.0052 (3)
S2	0.0299 (3)	0.0254 (3)	0.0259 (3)	−0.0017 (3)	0.0003 (3)	0.0027 (3)
O1	0.0372 (10)	0.0418 (11)	0.0361 (12)	0.0039 (8)	−0.0012 (8)	0.0105 (10)
O2	0.0464 (12)	0.0381 (11)	0.0412 (13)	−0.0101 (9)	−0.0084 (9)	0.0077 (10)
O3	0.0737 (13)	0.0331 (11)	0.0468 (14)	0.0164 (11)	0.0289 (13)	0.0041 (10)
O4	0.0434 (9)	0.0365 (10)	0.0302 (10)	−0.0086 (11)	−0.0086 (8)	0.0029 (9)
O5	0.0395 (11)	0.0529 (13)	0.0381 (12)	0.0061 (9)	0.0154 (9)	0.0089 (10)
O6	0.0417 (10)	0.0255 (10)	0.0392 (12)	0.0007 (8)	−0.0055 (9)	0.0042 (9)
O1W	0.0457 (11)	0.0422 (12)	0.0667 (16)	0.0074 (9)	0.0137 (11)	−0.0032 (12)
O2W	0.0438 (10)	0.0432 (13)	0.0420 (13)	0.0003 (9)	−0.0116 (9)	0.0002 (10)
O3W	0.0486 (12)	0.0399 (12)	0.0765 (19)	0.0157 (10)	0.0014 (11)	−0.0116 (12)
O4W	0.036 (2)	0.094 (4)	0.075 (6)	0.011 (2)	−0.010 (5)	−0.009 (5)
O4W'	0.036 (2)	0.094 (4)	0.075 (6)	0.011 (2)	−0.010 (5)	−0.009 (5)
O5W	0.0504 (12)	0.0479 (14)	0.088 (2)	0.0178 (11)	0.0124 (12)	0.0176 (14)
C1	0.0272 (12)	0.0259 (13)	0.0287 (15)	0.0009 (10)	0.0038 (10)	0.0067 (11)
C2	0.0434 (15)	0.0261 (13)	0.0237 (15)	0.0025 (11)	−0.0002 (11)	−0.0010 (10)
C3	0.0439 (14)	0.0294 (13)	0.0187 (13)	−0.0003 (11)	−0.0009 (12)	0.0045 (12)
C4	0.0233 (10)	0.0288 (13)	0.0207 (12)	−0.0010 (10)	0.0003 (9)	−0.0016 (12)
C5	0.0251 (11)	0.0284 (13)	0.0238 (13)	0.0010 (11)	0.0001 (9)	0.0009 (11)
C6	0.0339 (14)	0.0284 (14)	0.0361 (17)	−0.0007 (11)	−0.0043 (12)	−0.0028 (12)
C7	0.0439 (16)	0.0365 (17)	0.0309 (16)	0.0010 (14)	−0.0008 (13)	−0.0134 (13)
C8	0.0399 (14)	0.0418 (16)	0.0222 (15)	0.0024 (12)	−0.0030 (11)	−0.0032 (12)
C9	0.0228 (11)	0.0304 (14)	0.0248 (14)	0.0027 (10)	0.0019 (10)	−0.0005 (11)
C10	0.0335 (13)	0.0333 (14)	0.0202 (14)	0.0016 (11)	0.0016 (10)	0.0036 (11)

### Geometric parameters (Å, °)

**Table:**

Sr1—O1	2.737 (2)	O3W—H32	0.8400
Sr1—O2	2.721 (2)	O4W—H41	0.8400
Sr1—O3^i^	2.583 (2)	O4W—H42	0.8400
Sr1—O4^ii^	2.5352 (19)	O4W'—H43	0.8400
Sr1—O1W	2.641 (2)	O4W'—H44	0.8401
Sr1—O2W	2.562 (2)	O5W—H51	0.8430
Sr1—O3W	2.500 (2)	O5W—H52	0.8433
Sr1—O4W	2.585 (14)	C1—C10	1.353 (4)
Sr1—O4W'	2.542 (15)	C1—C2	1.413 (4)
S1—O1	1.448 (2)	C2—C3	1.363 (4)
S1—O3	1.448 (2)	C2—H2	0.9300
S1—O2	1.458 (2)	C3—C4	1.422 (4)
S1—C1	1.766 (3)	C3—H3	0.9300
S2—O5	1.450 (2)	C4—C5	1.415 (4)
S2—O4	1.4526 (19)	C4—C9	1.427 (3)
S2—O6	1.4550 (19)	C5—C6	1.382 (4)
S2—C5	1.767 (3)	C6—C7	1.397 (4)
O3—Sr1^iii^	2.583 (2)	C6—H6	0.9300
O4—Sr1^iv^	2.5352 (19)	C7—C8	1.366 (4)
O1W—H11	0.8401	C7—H7	0.9300
O1W—H12	0.8399	C8—C9	1.409 (4)
O2W—H21	0.8399	C8—H8	0.9300
O2W—H22	0.8399	C9—C10	1.420 (4)
O3W—H31	0.8401	C10—H10	0.9300
			
O3W—Sr1—O4^ii^	97.84 (7)	S1—O3—Sr1^iii^	150.00 (13)
O3W—Sr1—O4W'	75.6 (3)	S2—O4—Sr1^iv^	149.83 (12)
O4^ii^—Sr1—O4W'	88.3 (3)	Sr1—O1W—H11	109.5
O3W—Sr1—O2W	73.44 (7)	Sr1—O1W—H12	109.5
O4^ii^—Sr1—O2W	76.65 (7)	H11—O1W—H12	109.5
O4W'—Sr1—O2W	143.1 (2)	Sr1—O2W—H21	109.5
O3W—Sr1—O3^i^	146.15 (7)	Sr1—O2W—H22	109.5
O4^ii^—Sr1—O3^i^	82.38 (7)	H21—O2W—H22	109.5
O4W'—Sr1—O3^i^	70.6 (3)	Sr1—O3W—H31	109.5
O2W—Sr1—O3^i^	137.99 (7)	Sr1—O3W—H32	109.5
O3W—Sr1—O4W	67.3 (3)	H31—O3W—H32	109.5
O4^ii^—Sr1—O4W	80.5 (3)	Sr1—O4W—H41	109.9
O4W'—Sr1—O4W	12.6 (3)	Sr1—O4W—H42	109.6
O2W—Sr1—O4W	130.8 (2)	H41—O4W—H42	108.4
O3^i^—Sr1—O4W	79.5 (3)	Sr1—O4W—H43	114.0
O3W—Sr1—O1W	140.76 (7)	Sr1—O4W'—H43	109.6
O4^ii^—Sr1—O1W	89.77 (7)	H41—O4W'—H43	109.5
O4W'—Sr1—O1W	143.4 (3)	Sr1—O4W'—H44	109.5
O2W—Sr1—O1W	71.03 (7)	H43—O4W'—H44	109.5
O3^i^—Sr1—O1W	72.92 (8)	H51—O5W—H52	107.9
O4W—Sr1—O1W	151.7 (3)	C10—C1—C2	120.8 (2)
O3W—Sr1—O2	92.01 (8)	C10—C1—S1	120.8 (2)
O4^ii^—Sr1—O2	158.59 (6)	C2—C1—S1	118.3 (2)
O4W'—Sr1—O2	75.8 (3)	C3—C2—C1	120.0 (3)
O2W—Sr1—O2	124.55 (7)	C3—C2—H2	120.0
O3^i^—Sr1—O2	78.91 (7)	C1—C2—H2	120.0
O4W—Sr1—O2	85.9 (3)	C2—C3—C4	121.5 (3)
O1W—Sr1—O2	94.57 (7)	C2—C3—H3	119.2
O3W—Sr1—O1	76.18 (7)	C4—C3—H3	119.2
O4^ii^—Sr1—O1	149.74 (6)	C5—C4—C3	124.3 (2)
O4W'—Sr1—O1	117.8 (4)	C5—C4—C9	118.2 (2)
O2W—Sr1—O1	73.20 (7)	C3—C4—C9	117.5 (2)
O3^i^—Sr1—O1	119.14 (8)	C6—C5—C4	120.5 (2)
O4W—Sr1—O1	122.0 (4)	C6—C5—S2	117.7 (2)
O1W—Sr1—O1	78.10 (6)	C4—C5—S2	121.72 (19)
O2—Sr1—O1	51.35 (6)	C5—C6—C7	120.6 (3)
O1—S1—O3	113.78 (13)	C5—C6—H6	119.7
O1—S1—O2	108.91 (12)	C7—C6—H6	119.7
O3—S1—O2	112.83 (14)	C8—C7—C6	120.5 (3)
O1—S1—C1	107.58 (13)	C8—C7—H7	119.8
O3—S1—C1	105.46 (12)	C6—C7—H7	119.8
O2—S1—C1	107.94 (12)	C7—C8—C9	120.6 (3)
O5—S2—O4	112.99 (12)	C7—C8—H8	119.7
O5—S2—O6	111.86 (12)	C9—C8—H8	119.7
O4—S2—O6	110.92 (11)	C8—C9—C10	120.5 (3)
O5—S2—C5	106.59 (12)	C8—C9—C4	119.6 (2)
O4—S2—C5	107.54 (11)	C10—C9—C4	119.8 (2)
O6—S2—C5	106.53 (13)	C1—C10—C9	120.3 (2)
S1—O1—Sr1	99.67 (9)	C1—C10—H10	119.8
S1—O2—Sr1	100.05 (10)	C9—C10—H10	119.8
			
O3—S1—O1—Sr1	125.47 (11)	O3—S1—C1—C2	−42.8 (2)
O2—S1—O1—Sr1	−1.34 (13)	O2—S1—C1—C2	78.0 (2)
C1—S1—O1—Sr1	−118.10 (10)	C10—C1—C2—C3	−1.6 (4)
O3W—Sr1—O1—S1	104.94 (11)	S1—C1—C2—C3	176.90 (19)
O4^ii^—Sr1—O1—S1	−173.28 (9)	C1—C2—C3—C4	0.6 (4)
O4W'—Sr1—O1—S1	39.8 (3)	C2—C3—C4—C5	−178.8 (2)
O2W—Sr1—O1—S1	−178.50 (12)	C2—C3—C4—C9	1.2 (4)
O3^i^—Sr1—O1—S1	−42.42 (12)	C3—C4—C5—C6	179.6 (2)
O4W—Sr1—O1—S1	53.5 (3)	C9—C4—C5—C6	−0.5 (3)
O1W—Sr1—O1—S1	−104.96 (11)	C3—C4—C5—S2	1.7 (3)
O2—Sr1—O1—S1	0.87 (8)	C9—C4—C5—S2	−178.37 (17)
O1—S1—O2—Sr1	1.35 (13)	O5—S2—C5—C6	−110.1 (2)
O3—S1—O2—Sr1	−126.00 (12)	O4—S2—C5—C6	128.4 (2)
C1—S1—O2—Sr1	117.88 (11)	O6—S2—C5—C6	9.5 (2)
O3W—Sr1—O2—S1	−71.35 (11)	O5—S2—C5—C4	67.8 (2)
O4^ii^—Sr1—O2—S1	171.05 (13)	O4—S2—C5—C4	−53.7 (2)
O4W'—Sr1—O2—S1	−145.9 (3)	O6—S2—C5—C4	−172.64 (19)
O2W—Sr1—O2—S1	−0.13 (14)	C4—C5—C6—C7	0.8 (4)
O3^i^—Sr1—O2—S1	141.52 (12)	S2—C5—C6—C7	178.7 (2)
O4W—Sr1—O2—S1	−138.4 (3)	C5—C6—C7—C8	−1.1 (4)
O1W—Sr1—O2—S1	69.94 (11)	C6—C7—C8—C9	1.1 (4)
O1—Sr1—O2—S1	−0.87 (8)	C7—C8—C9—C10	−178.2 (2)
O1—S1—O3—Sr1^iii^	−82.0 (3)	C7—C8—C9—C4	−0.8 (4)
O2—S1—O3—Sr1^iii^	42.7 (3)	C5—C4—C9—C8	0.5 (4)
C1—S1—O3—Sr1^iii^	160.3 (3)	C3—C4—C9—C8	−179.6 (2)
O5—S2—O4—Sr1^iv^	23.0 (3)	C5—C4—C9—C10	177.9 (2)
O6—S2—O4—Sr1^iv^	−103.6 (2)	C3—C4—C9—C10	−2.2 (3)
C5—S2—O4—Sr1^iv^	140.3 (2)	C2—C1—C10—C9	0.6 (4)
O1—S1—C1—C10	13.9 (2)	S1—C1—C10—C9	−177.81 (19)
O3—S1—C1—C10	135.7 (2)	C8—C9—C10—C1	178.6 (2)
O2—S1—C1—C10	−103.5 (2)	C4—C9—C10—C1	1.3 (4)
O1—S1—C1—C2	−164.6 (2)		

Symmetry codes: (i) *x*+1/2, −*y*+3/2, −*z*+2; (ii) −*x*+1/2, −*y*+1, *z*−1/2; (iii) *x*−1/2, −*y*+3/2, −*z*+2; (iv) −*x*+1/2, −*y*+1, *z*+1/2.

### Hydrogen-bond geometry (Å, °)

**Table:**

*D*—H···*A*	*D*—H	H···*A*	*D*···*A*	*D*—H···*A*
O1w—H12···O2^iii^	0.84	2.25	2.809 (3)	124
O2w—H21···O5^v^	0.84	2.03	2.793 (3)	151
O2w—H22···O5w^vi^	0.84	1.95	2.763 (3)	164
O3w—H31···O6^vii^	0.84	2.09	2.829 (3)	147
O3w—H32···O5w^vi^	0.84	1.99	2.754 (3)	151
O5w—H51···O6^ii^	0.84	2.06	2.874 (3)	163
O5w—H52···O1w	0.84	2.02	2.831 (3)	160

Symmetry codes: (iii) *x*−1/2, −*y*+3/2, −*z*+2; (v) −*x*−1/2, −*y*+1, *z*−1/2; (vi) −*x*, *y*−1/2, −*z*+3/2; (vii) *x*+1/2, −*y*+1/2, −*z*+2; (ii) −*x*+1/2, −*y*+1, *z*−1/2.
